# Rapid, inexpensive, fingerstick, whole-blood, sensitive, specific, point-of-care test for anti-*Toxoplasma* antibodies

**DOI:** 10.1371/journal.pntd.0006536

**Published:** 2018-08-16

**Authors:** Joseph Lykins, Xuan Li, Pauline Levigne, Ying Zhou, Kamal El Bissati, Fatima Clouser, Martine Wallon, Florence Morel, Karen Leahy, Bouchra El Mansouri, Maryam Siddiqui, Nicole Leong, Morgan Michalowski, Erin Irwin, Perpetua Goodall, Mahmoud Ismail, Monica Christmas, El Bachir Adlaoui, Mohamed Rhajaoui, Amina Barkat, Hua Cong, Ian J. Begeman, Bo Shiun Lai, Despina G. Contopoulos-Ioannidis, Jose G. Montoya, Yvonne Maldonado, Raymund Ramirez, Cindy Press, Francois Peyron, Rima McLeod

**Affiliations:** 1 Pritzker School of Medicine, University of Chicago, Chicago, Illinois, United States of America; 2 Rush Medical College, Rush University, Chicago, Illinois, United States of America; 3 Institut de Parasitologie et de Mycologie Médicale Hôpital de la Croix Rousse, Lyon, France; 4 Department of Ophthalmology and Visual Sciences, University of Chicago, Chicago, Illinois, United States of America; 5 Department of Obstetrics and Gynecology, University of Chicago, Chicago, Illinois, United States of America; 6 Institut National d’Hygiène, Rabat, Morocco; 7 Équipe de recherche en santé et nutrition du couple mère enfant, Faculté de Médecine et de Pharmacie de Rabat, Université Mohammed V, Rabat, Morocco; 8 Department of Pediatrics, Division of Infectious Diseases, Stanford University School of Medicine, Stanford, California, United States of America; 9 Palo Alto Medical Foundation Toxoplasma Serology Laboratory, Palo Alto, California, United States of America; 10 Department of Medicine, Division of Infectious Diseases and Geographic Medicine, Stanford University School of Medicine, Stanford, California, United States of America; 11 Department of Health Research and Policy, Stanford University School of Medicine, Stanford, California, United States of America; 12 Section of Infectious Diseases, Department of Pediatrics, Institute of Genomics, Genetics, and Systems Biology, Global Health Center, Toxoplasmosis Center, CHeSS, The College, University of Chicago, Chicago, Illinois, United States of America; University of Iowa, UNITED STATES

## Introduction

Toxoplasmosis causes substantial morbidity and mortality on a global scale [1]. This disease can be severe. Vertical transmission from mother to fetus occurs from primary acute infection during gestation. Congenital infection may result in chorioretinitis, hydrocephalus, epilepsy, and death [2]. Serologic screening during gestation allows early antenatal detection and rapid treatment initiation, with economic and patient outcome benefits [3–5]. However, conventional serologic screening can be cost prohibitive. Infrastructure, including electricity and equipment for sample processing, may be unavailable. Point-of-care (POC) tests detecting *Toxoplasma gondii* infection offer solutions, potentially addressing cost concerns and leading to better clinical outcomes through improved access to screening. Testing performance of a lateral flow immunochromatography-based *Toxoplasma* ICT IgG–IgM test for combined detection of *Toxoplasma*-specific IgG and IgM has been previously described using serum samples from the National Collaborative Chicago-Based Congenital Toxoplasmosis Study (NCCCTS) and other cohorts [6–8]. Herein, we test whether a comparably performing whole-blood–variant test (designated “BK”) could perform with high sensitivity and specificity, obviating the need for venipuncture and sample processing infrastructure and making an efficient, low-cost POC test.

## Methods

### Volunteer recruitment and obtaining samples

Samples were obtained from consenting United States individuals, including seropositive individuals affiliated with the NCCCTS [4,6] and obstetrical patients in Chicago, and obstetrical patients in Morocco (Table 1). Further details of participant characteristics and recruitment can be found in S1 Methods. No incentives were provided for participation. Each person of unknown serologic status underwent venipuncture, and status was confirmed either with ARCHITECT Toxo-IgG and IgM system in the Lyon, France Reference Laboratory (n = 95 persons), or for Moroccan patients with Platelia Toxo IgG and IgM system (n = 39).

**Table 1:** Breakdown of participant characteristics.

	Patients	Samples	Serology already known	Serum and POC tested concurrently	United States patients	Morocco patients
Seronegative* (IgG and IgM negative in reference tests)	105	143**	8	135	99	6
Seropositive* (IgG and/or IgM positive in reference tests)	100	101***	64	37	67	33

*For sera samples, pink-line and black-line POC tests were 100% concordant. For whole-blood samples, we (FP) had found that the pink-line test kit was not useful when testing whole blood, as the lighter indicator was obscured by the whole blood.

**19 pregnant women were tested a total of three times each in an ongoing pilot program for screening during gestation.

***One person was tested twice to confirm accuracy of test across and at differing times.

**Abbreviation:** POC, point of care.

### Fingerstick protocol, whole blood-POC/serum-variant test kit, and comparison to serum-variant test kit

Participants provided whole blood via fingerstick (S1 Movie). Participants’ fingers were compressed, suffusing the tip, and cleaned with an alcohol wipe. A standard lancet was used for fingerstick. Capillary tubes allowed collection of 30 *μ*L of blood, which was directly applied to the *Toxoplasma* ICT IgG–IgM–BK (LDBIO) test, followed by application of four drops of buffer, provided in the kit. Tests were interpreted at 20–30 minutes by individuals performing tests and photographed for later interpretation by two individuals unaware of the subjects’ identity and serologic status.

Earlier, we described 100% sensitivity and specificity of a pink-line, serum-variant of this POC test [6]. Given its high performance, we compared the results of whole-blood black-line POC tests and serum-variant testing performed concurrently for 78 persons.

### Ethics

All participants provided written, informed consent. This study was performed in accordance with the rules and regulations of the University of Chicago Institutional Review Board (protocol #8793) and/or IRB approval in Morocco.

## Results

### Obtaining samples

**Fig 1:**
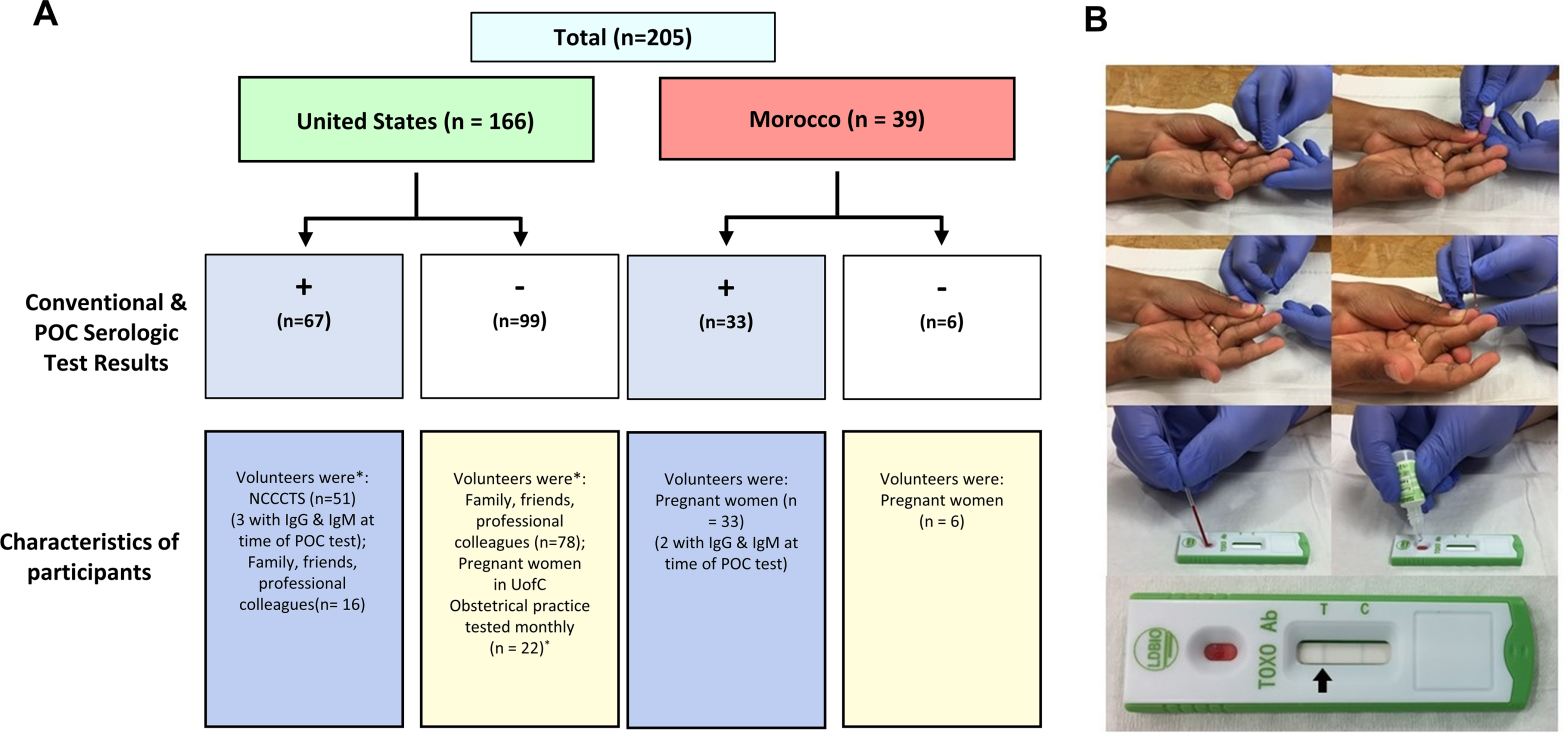
A) Study participant composition in United States and Morocco. One seropositive individual was tested via POC test twice but is included in this number only once. 19 pregnant women were tested a total of three times each as part of a pilot gestational screening program. All were seronegative. Volunteers learned of the study through word of mouth—sometimes when friends or family members knew of an affected child and asked to participate—or were formally recruited in an obstetrical practice. No one complained or mentioned medical problems of any type other than as related to toxoplasmosis among those who were seropositive from the NCCCTS or pregnancy, although there was no health questionnaire or physical examination as part of this study. B) Performing the *Toxoplasma* ICT IgG–IgM Test. The site of the fingerstick is cleansed with an alcohol wipe and pricked with a lancet. Blood is collected via capillary tube and applied to the test kit. Four drops of eluent are then applied. Test can be interpreted in 20–30 minutes. The black arrow adjacent to the “T” indicates the presence of antibodies against *T*. *gondii*. NCCCTS, National Collaborative Chicago-Based Congenital Toxoplasmosis Study; POC, point of care; UofC, University of Chicago.

A total of 205 persons (Fig 1A, Table 2) (244 samples) had their serologic status for *T*. *gondii* assessed using the whole blood-variant test and confirmed using their NCCCTS records (n = 71), or concurrent standard laboratory testing (n = 134). Nineteen seronegative pregnant women had fingerstick and venipuncture performed each month for the first three months and three more for the first month (two with pregnancy loss) in an ongoing pilot gestational screening program, adding 41 samples to make 244 total samples. Overall, 101 samples proved seropositive, including five from acutely infected individuals who had IgM/IgG antibodies against *T*. *gondii*, while 143 were seronegative. Seropositive is defined here as having detectable anti-*Toxoplasma* IgG and/or IgM. For a small subset of pediatric patients (n = 7, ages three weeks to seven years) who were undergoing venipuncture as part of routine clinical care, whole blood was obtained from the needle tip to avoid a second, potentially distressing fingerstick.

**Table 2:** Patient totals by country.

Country	Seronegative samples tested (by POC and reference test)	Seropositive samples tested (by POC and reference test)	Acute samples (IgG + IgM tested with reference test)
United States	137	68	3^#^
Morocco	6	33	2^#^
Total	143	101	5^#^

^#^Also included in seropositive person totals. The US persons with IgM antibodies were sometimes followed over time, and all had adjunctive testing such as anti-*T*. *gondii* IgA, differential agglutination and avidity tests [2].

**Abbreviation:** POC, point of care.

### Sensitivity and specificity of whole blood/serum variant and comparability to serum-variant *Toxoplasma* ICT IgG–IgM POC test

The whole-blood test proved highly sensitive and specific, with a sensitivity of 100% (95% confidence interval [CI]: 96.41%–100%) and specificity of 100% (95% CI: 97.45%–100%) (Table 3). Whole-blood, serum-variant, and reference testing demonstrated 100% concordance. Of note, individuals with lower levels of anti-*Toxoplasma* antibodies infected at remote times and with lower titers were positive in the POC test in the range detected by gold-standard tests (S1 Table).

**Table 3:** Test results, sensitivity, specificity, and confidence intervals.

Test Result	Seropositive (reference test)	Seronegative (reference test)
Seropositive (whole blood–POC Test)	101^+^	0
Seronegative (whole blood–POC Test)	0^++^	143
Test parameter	Diagnostic performance	95% Confidence Interval
Sensitivity	100%	96.41%–100%^++^
Specificity	100%	97.45%–100%^++^

^+^Confirmatory tests allow a provider to distinguish acute from chronic infection. Acute infection during gestation requires anti-*Toxoplasma* medicines to prevent or reduce vertical transmission. Chronic infection requires no further testing during gestation. The first test should be performed by 12 weeks gestation to facilitate distinction of acute and chronic infection [1].

^++^A very faint grey *Toxoplasma* (T) band was noted transiently for one person tested prospectively. This band was not visible when photographed at 20 and 30 minutes. In accordance with the manufacturer’s instructions, this result was designated “equivocal or indeterminate.” This patient was determined, through confirmatory testing, to have been negative for *T*. *gondii* antibodies, and subsequent testing with the POC test failed to show any *Toxoplasma* band. Any such equivocal result requires back-up testing, as does any first positive result for a pregnant woman.

**Abbreviation:** POC, point of care.

### Feasibility of fingerstick as testing modality

No participant refused a second fingerstick, although they were informed they could. Participants did not report significant discomfort associated with fingerstick. Patients and providers enthusiastically accepted the monthly gestational screening program.

## Discussion

Point-of-care testing for *Toxoplasma* infection has the potential to markedly change clinical approach to this infection by detecting seroconversion using small volumes of whole blood—obviating venipuncture and expensive equipment for serum separation—and performing with high sensitivity and specificity. Advantages and disadvantages are discussed in Box 1.

**Box 1. Advantages and disadvantages of the whole-blood–variant *Toxoplasma* ICT IgG-IgM test**

Advantages:

- True POC test, useful in a standard obstetrician’s office and less invasive than conventional serologic testing (requiring materials to perform venipuncture, electricity and a centrifuge for serum separation, and skilled technicians). POC testing has the potential to significantly expand access to screening during gestation for this serious, potentially fatal infection, with spillover benefit in facilitating screening programs for other congenital infections and improvements in maternal fetal health and well-being and care. Our demonstration of the high performance of this whole-blood POC test and the test’s strong functionality at the POC, which has not been previously demonstrated, provide the proof of principle of its potential utility and widespread applicability in clinical settings. This new test also fulfills the World Health Organization criteria for the ideal POC test (affordable, sensitive, specific, user friendly, rapid/robust, equipment-free, and deliverable to users).
- Performance and interpretation of test take less than two minutes of operator time with results available to be interpreted within 20–30 minutes, facilitating appropriate clinical intervention so that it is ideal for following those who are seronegative to detect seroconversion.
- No need for expensive equipment/electricity, decreasing cost and enhancing utility, especially in developing countries.

Disadvantages:

- Requires initial, alternative testing for those who are seropositive that distinguishes IgG from IgM and tests for avidity of antibody for those who have *T*. *gondii*-specific IgM. It also requires follow-up testing for seroconversion when used for screening during gestation.
- Requires a fingerstick, which occasionally may need to be performed more than once due to technical difficulties.
- Requires materials (alcohol wipe, lancet, and bandage) and training to perform and interpret test.

While the cost effectiveness of gestational screening and antenatal treatment in the US is demonstrated for a wide range of parameters [3], it was suggested that screening could not be financially viable in the US [9]. Nevertheless, there is robust evidence demonstrating cost effectiveness in other countries [10]. Adoption of this POC test would substantially reduce costs further and require significantly less infrastructure than conventional testing. Value also arises from bringing pregnant women into care, ensuring screening for other preventable and treatable conditions, particularly in developing countries [6]. This POC test cannot distinguish seropositivity for IgG and IgM and, therefore, cannot distinguish acute from chronic infection. A positive POC test prior to 12 weeks of gestation requires further confirmatory testing. Confirmatory testing and guidance concerning additional testing and treatment will benefit patients and their physicians. In countries with high seroprevalence, this benefit would be particularly important. This POC test demonstrated superb diagnostic performance in countries in the developed and developing world, with genetically distinct patients and parasites. The serum-variant test also has exceptional ability to identify samples in which both *Toxoplasma*-specific IgG and IgM, or IgG or IgM alone, are present. It does not identify natural IgM antibodies, which are false positives [8]. For initial testing before 12 weeks gestation, optimal testing would accurately test for IgM and IgG separately, allowing rapid distinction between recently acquired and chronic infection. Initial, inexpensive tests multiplexed for multiple congenital infections could further improve prenatal screening. Ultimately, it is accurate, well-validated POC testing, rather than a specific company’s test, that can reduce morbidity and mortality from congenital toxoplasmosis and most benefit patients and their families.

## Supporting information

S1 Methods Detailed description of participant characteristics and recruitment. (DOCX)

S1 Table Serologic results for all included participants in the United States (A) and Morocco (B).
(DOCX)

S1 Movie Fingerstick protocol with details of test performance. (MP4)
