# Supplementary material for: Rapid, inexpensive, fingerstick, whole-blood, sensitive, specific, point-of-care test for anti-Toxoplasma antibodies
Source: PLoS Negl Trop Dis. 2018 Aug 16;12(8):e0006536. doi: 10.1371/journal.pntd.0006536 (PMC6095485; doi:10.1371/journal.pntd.0006536)
Supplement: S1 Methods — (DOCX) [file pntd.0006536.s001.docx]

**Supporting Information**

**Supplemental Methods**

**Rapid, inexpensive, fingerstick, whole blood, sensitive, specific, point-of-care test for anti-*Toxoplasma* antibodies**

Joseph Lykins ^1^, Xuan Li ^2^, Pauline Levigne ^3^, Ying Zhou ^4^, Kamal El Bissati ^4^, Fatima Clouser ^4^, Martine Wallon ^3^, Florence Morel ^3^, Karen Leahy ^5^, Bouchra El Mansouri ^6^, Maryam Siddiqui ^5^, Nicole Leong ^5^, Morgan Michalowski ^5^, Erin Irwin ^5^, Perpetua Goodall ^5^, Mahmoud Ismail ^5^, Monica Christmas ^5^, El Bachir Adlaoui ^6^, Mohamed Rhajaoui ^6^, Amina Barkat ^7^, Hua Cong ^4^, Ian J. Begeman ^4^, Bo Shiun Lai ^4^, Despina G. Contopoulos-Ioannidis ^8^, Jose G. Montoya ^9,10^, Yvonne Maldonado ^8,11^, Raymund Ramirez ^9^, Cindy Press ^9^, Francois Peyron ^3^, Rima McLeod ^1,12*^

^1^ Pritzker School of Medicine, University of Chicago, Chicago, Illinois, USA

^2^ Rush Medical College, Rush University, Chicago, Illinois, USA

^3^ Institut de Parasitologie et de Mycologie Médicale Hôpital de la Croix Rousse, 103 grande rue de la Croix Rousse, 69317, Lyon, France

^4^ Department of Ophthalmology and Visual Sciences, University of Chicago, Chicago, Illinois, USA

^5^ Department of Obstetrics and Gynecology, University of Chicago, Chicago, Illinois, USA

^6^ Institut National d’Hygiène, Rabat, Morocco

^7^ Équipe de recherche en santé et nutrition du couple mère enfant, Faculté de Médecine et de Pharmacie de Rabat, Université Mohammed V, Rabat, Morocco

^8^ Department of Pediatrics, Division of Infectious Diseases, Stanford University School of Medicine, Stanford, California, USA

^9^ Palo Alto Medical Foundation Toxoplasma Serology Laboratory, Palo Alto, California, USA

^10^ Department of Medicine, Division of Infectious Diseases and Geographic Medicine, Stanford University School of Medicine, Stanford, California, USA

^11^ Department of Health Research and Policy, Stanford University School of Medicine, Stanford, California, USA

^12^ Section of Infectious Diseases, Department of Pediatrics, Institute of Genomics, Genetics, and Systems Biology, Global Health Center, Toxoplasmosis Center, CHeSS, The College, University of Chicago, Chicago, Illinois, USA

*Corresponding author

E-mail: rmcleod@uchicago.edu

**Supplemental Methods:**

**Participant Characteristics and Recruitment**

U.S. samples were obtained from the following volunteers: seropositive individuals, within the NCCCTS, and their families; randomly selected obstetrics patients from the University of Chicago, as well as healthy volunteers recruited for participation to whom the study and its purpose was described. All volunteers were asked if they would be willing to come to the University of Chicago to provide POC samples. Samples were also obtained from otherwise healthy, randomly selected pregnant women who elected to be screened in Morocco. No incentives were provided for participation. Each person of unknown serologic status underwent venipuncture, and status was confirmed either with the ARCHITECT Toxo-IgG and IgM system in Lyon, France or via the Platelia ^TM^ system in Rabat, Morocco. For some persons, serologic test results were available from remote testing at a reference laboratory, and when they were known to be positive, for example at the time of delivery of a congenitally infected infant, testing was not repeated. Word of mouth recruited volunteers (including family and friends) to participate in the study. We were not made aware of any illness in the persons who volunteered, but we did not take a detailed medical history, nor perform a thorough medical examination.
