# Supplementary material for: Rapid, inexpensive, fingerstick, whole-blood, sensitive, specific, point-of-care test for anti-Toxoplasma antibodies
Source: PLoS Negl Trop Dis. 2018 Aug 16;12(8):e0006536. doi: 10.1371/journal.pntd.0006536 (PMC6095485; doi:10.1371/journal.pntd.0006536)
Supplement: S1 Table — Serologic results for all included participants in the United States (A) and Morocco (B). (DOCX) [file pntd.0006536.s002.docx]

**Supporting Information**

**Supplemental Table S1**

**Rapid, inexpensive, fingerstick, whole blood, sensitive, specific, point-of-care test for anti-*Toxoplasma* antibodies**

Joseph Lykins ^1^, Xuan Li ^2^, Pauline Levigne ^3^, Ying Zhou ^4^, Kamal El Bissati ^4^, Fatima Clouser ^4^, Martine Wallon ^3^, Florence Morel ^3^, Karen Leahy ^5^, Bouchra El Mansouri ^6^, Maryam Siddiqui ^5^, Nicole Leong ^5^, Morgan Michalowski ^5^, Erin Irwin ^5^, Perpetua Goodall ^5^, Mahmoud Ismail ^5^, Monica Christmas ^5^, El Bachir Adlaoui ^6^, Mohamed Rhajaoui ^6^, Amina Barkat ^7^, Hua Cong ^4^, Ian J. Begeman ^4^, Bo Shiun Lai ^4^, Despina G. Contopoulos-Ioannidis ^8^, Jose G. Montoya ^9,10^, Yvonne Maldonado ^8,11^, Raymund Ramirez ^9^, Cindy Press ^9^, Francois Peyron ^3^, Rima McLeod ^1,12*^

^1^ Pritzker School of Medicine, University of Chicago, Chicago, Illinois, USA

^2^ Rush Medical College, Rush University, Chicago, Illinois, USA

^3^ Institut de Parasitologie et de Mycologie Médicale Hôpital de la Croix Rousse, 103 grande rue de la Croix Rousse, 69317, Lyon, France

^4^ Department of Ophthalmology and Visual Sciences, University of Chicago, Chicago, Illinois, USA

^5^ Department of Obstetrics and Gynecology, University of Chicago, Chicago, Illinois, USA

^6^ Institut National d’Hygiène, Rabat, Morocco

^7^ Équipe de recherche en santé et nutrition du couple mère enfant, Faculté de Médecine et de Pharmacie de Rabat, Université Mohammed V, Rabat, Morocco

^8^ Department of Pediatrics, Division of Infectious Diseases, Stanford University School of Medicine, Stanford, California, USA

^9^ Palo Alto Medical Foundation Toxoplasma Serology Laboratory, Palo Alto, California, USA

^10^ Department of Medicine, Division of Infectious Diseases and Geographic Medicine, Stanford University School of Medicine, Stanford, California, USA

^11^ Department of Health Research and Policy, Stanford University School of Medicine, Stanford, California, USA

^12^ Section of Infectious Diseases, Department of Pediatrics, Institute of Genomics, Genetics, and Systems Biology, Global Health Center, Toxoplasmosis Center, CHeSS, The College, University of Chicago, Chicago, Illinois, USA

*Corresponding author

E-mail: rmcleod@uchicago.edu

Table S1. Serologic Results

A. United States

| **Year of POC Test** | **Result of POC Test ^a^** | **Participant**  **Number** | **Year of Conventional Test ^b^** | **Reciprocal IgG ^c^** | **IgM ^d^** | **IgA ^e^** | **IgE** | **AC/HS** | **Avidity ^f^** | **Other** |
| --- | --- | --- | --- | --- | --- | --- | --- | --- | --- | --- |
| 2017 | Positive | 1 | 2015 | 96 | 0.03 |  |  |  |  |  |
| 2017 | Positive | 2 | 1977 | 4096 | IgM IFA: 320 | ND | ND | ND | ND | n/a |
| 2017 | Positive | 3 | 2017 | 4.10 ^*^ | 0.08 |  |  |  |  |  |
| 2017 | Positive | 4 | 2015 | 32 | ELISA: Negative |  |  |  |  |  |
| 2017 | Positive | 5 | 1992 | 512 | ISAGA: 3 | 1.2 |  |  |  |  |
| 2017 | Positive | 6 | 1992 | 4096 | ELISA: 5.2 | 6.9 |  | 1600/3200 |  |  |
| 2017 | Positive | 7 | 1991 | 128 | ELISA: 0 |  |  |  |  |  |
| 2017 | Positive | 8 | 2000 | 32000 | ISAGA: Positive | 28 |  |  |  |  |
| 2017 | Positive | 9 | 2000 | 16000 | ELISA: 9.1 | 9.5 | Positive | 1600/3200 |  |  |
| 2017 | Positive | 10 | Newborn Screen, Umass | 0.651 ^*^ | 0.338 |  |  |  |  |  |
| 2017 | Positive | 11 | 2009 | 97.9 IU/mL ^*^ | <0.9 |  |  |  |  |  |
| 2017 | Positive | 12 | 1976 | 256 | < 1.6 IU/mL | ND | ND | ND | ND | n/a |
| 2017 | Positive | 13 | 2000 | 512 | ISAGA: 0 |  | Negative |  |  |  |
| 2017 | Positive | 14 | 2000 | 8000 | ELISA: 4.4 | 1.2 | Positive | 1600/1600 |  |  |
| 2017 | Positive | 15 | 1987 | 4096 | ELISA: 9.2 |  |  |  |  | Agglutination: 16000 |
| 2017 | Positive | 16 | 1987 | 2048 | ELISA: 3.5 |  |  |  |  | Agglutination: 20480 |
| 2017 | Positive | 17 | 2012 | 2048 | ELISA: 4.7 | 26 | Equivocal | 800/400 | Low |  |
| 2017 | Positive | 18 | 2017 | 10.70 ^*^ | 0.70 |  |  |  |  |  |
| 2017 | Positive | 19 | 2017 | 52.70 ^*^ | 0.53 |  |  |  |  |  |
| 2017 | Positive | 20 | 2014 | 4096 | ELISA: 7.1 |  |  | >1600/800 |  |  |
| 2017 | Positive | 21 | 1999 | 64000 | ISAGA: 12 | 28 |  |  |  |  |
| 2017 | Positive | 22 | 2000 | 8000 | ELISA: 2.3 | 9.1 | Equivocal | 1600/3200 |  |  |
| 2017 | Positive | 23 | 2007 | 8000 | ELISA: 5.5 | 7.2 |  | 1600/3200 |  |  |
| 2017 | Positive | 24 | 2007 | 8000 | ELISA: 3.4 | 0.4 | Positive | 50/3200 |  |  |
| 2017 | Positive | 25 | 2007 | 4096 | ELISA: 0.2 | 1 | Negative | 200/3200 |  |  |
| 2017 | Positive | 26 | 2008 | 512 | 0.1 |  |  | 100/<100, E | ND |  |
| 2017 | Positive | 27 | 2009 | 8000 | ELISA: 3 | 3.5 | Negative | 1600/3200 | Equivocal |  |
| 2017 | Positive | 28 | 2010 | 8000 | ISAGA: Positive | 11 |  |  |  |  |
| 2017 | Positive | 29 | 2010 | 8000 |  |  |  |  |  |  |
| 2017 | Positive | 30 | 2013 | 8000 | ISAGA: Positive | 2.1 |  |  |  |  |
| 2017 | Positive | 31 | 2013 | 8000 | ELISA: 5 |  |  |  |  |  |
| 2017 | Positive | 32 | 2016 | 8000 | ISAGA: Positive | 1.3 |  |  |  |  |
| 2017 | Positive | 33 | 2016 | 2048 | ELISA: 2.6 | Neg | Neg | >1600/>3200 | Equivocal |  |
| 2017 | Positive | 34 | 2012 | 10.5 IU/mL ^*^ |  |  |  |  |  |  |
| 2017 | Positive | 35 | 1998 | 128 | ELISA: 0 | 0.5 | Negative | 50/400 |  |  |
| 2017 | Positive | 36 | 1998 | 256 | ELISA: 0 | 1 | Negative | 50/400 |  |  |
| 2017 | Positive | 37 | 2013 | 15.0 IU/mL ^*^ |  |  |  |  |  |  |
| 2017 | Positive | 38 | 2008 | 32000 | ELISA: 6.5 | 11.2 | Positive | 1600/3200 |  |  |
| 2017 | Positive | 39 | 2017 | 2048 | ELISA: 11 | 6 | Positive | 400/400 |  |  |
| 2017 | Positive | 40 | 2017 | 2048 |  |  |  |  |  |  |
| 2017 | Positive | 41 | 1996 | 4096 |  |  |  |  |  |  |
| 2017 | Positive | 42 | 2014 | 8000 | ISAGA: Positive | 10.8 |  |  |  |  |
| 2017 | Positive | 43 | 1998 | 2048 | ELISA: 1.6 | 0.2 | 1.6 | 50/3200 |  | IgI: 0 |
| 2017 | Positive | 44 | 2017 | 27.10 ^*^ | 3.69 |  |  |  |  |  |
| 2017 | Positive | 45 | 1986 | 2048 | ELISA: 0 |  |  |  |  |  |
| 2017 | Positive | 46 | 1986 | 8000 | ELiSA: 4.5 |  |  |  |  | Agglutination: 80000 |
| 2017 | Positive | 47 | 2009 | >2500 IU/mL^*^ | 0.15 |  |  |  |  |  |
| 2017 | Positive | 48 | 2017 | 4.10 ^*^ | 0.08 |  |  |  |  |  |
| 2017 | Positive | 49 | 2017 | 4.50 ^*^ | 0.05 |  |  |  |  |  |
| 2017 | Positive | 50 | 2017 | 26.20 ^*^ | 0.40 |  |  |  |  |  |
| 2017 | Positive | 51 | 2017 | 23.50 ^*^ | 0.09 |  |  |  |  |  |
| 2017 | Positive | 52 | 2011 | 8000 | ELISA: 4 | 6.3 | Positive | 1600/3200 |  |  |
| 2017 | Positive | 53 | 2011 | 32000 | ISAGA: Positive | > 11.0 |  |  |  |  |
| 2017 | Positive | 54 | 2017 | 4.70 ^*^ | 0.06 |  |  |  |  |  |
| 2017 | Positive | 55 | 2001 | 80 IU/mL ^*^ | 12 |  |  | HS 16 |  | In Paris |
| 2017 | Positive | 56 | 2001 | 80 IU/mL ^*^ | ISAGA: 12 | 8 |  |  |  | In Paris |
| 2018 | Positive | 57 | 2017 | 2048 | ISAGA: Positive | 0 |  |  |  |  |
| 2018 | Positive | 58 | 2017 | 2048 | ELISA: 2 | 0.3 | Positive | 800/1600 |  |  |
| 2018 | Positive | 59 | 2018 | 2048 | ISAGA: Negative | 0.2 |  |  |  |  |
| 2018 | Positive | 60 | 2018 | 0.175 ^*^ | ELISA: 2.3 | 0.7 | Negative | 200/400 |  |  |
| 2018 | Positive | 61 | 2018 | 19.00 ^*^ | 0.15 |  |  |  |  |  |
| 2018 | Positive | 62 | 2017 | 512 | ISAGA: Negative | 0 |  |  |  |  |
| 2018 | Positive | 63 | 2017 | 8000 | ELISA: 0.8 |  |  | >1600/>3200 | Equivocal |  |
| 2018 | Positive | 64 | 2012 | 8000 | 0 |  |  |  |  |  |
| 2018 | Positive | 65 | 2012 | 2048 | 1.6 |  |  |  | High |  |
| 2018 | Positive | 66 | 1991 | 16 | ELISA: 0 | 0.01 | Negative | <50/800 |  |  |
| 2018 | Positive | 67 | 1997 | 12000 |  |  |  |  |  |  |
| 2017-2018 | Negative | 68-168 | 2017-2018 | < 1.6 IU/mL | < 0.5 IU/mL | ND | ND | ND | ND | N/A |

B. Morocco

| **Year of POC Test** | **Result of POC Test ^a^** | **Participant**  **Number** | **Date of Conventional Test ^b^** | **Reciprocal IgG ^c^** | **IgM ^d^** | **IgA ^e^** | **IgE** | **ACHS** | **Avidity ^f^** | **Other** |
| --- | --- | --- | --- | --- | --- | --- | --- | --- | --- | --- |
| 2017 | Positive | 1 | 11/7/2017 | 148 ^*^ | NEG | ND | ND | ND | ND | N/A |
| 2017 | Positive | 2 | 11/7/2017 | 240 ^*^ | NEG | ND | ND | ND | ND | N/A |
| 2017 | Positive | 3 | 11/8/2017 | 52 ^*^ | NEG | ND | ND | ND | ND | N/A |
| 2017 | Positive | 4 | 11/8/2017 | 110 ^*^ | NEG | ND | ND | ND | ND | N/A |
| 2017 | Positive | 5 | 11/8/2017 | 130 ^*^ | NEG | ND | ND | ND | ND | N/A |
| 2017 | Positive | 6 | 11/9/2017 | 100 ^*^ | NEG | ND | ND | ND | ND | N/A |
| 2017 | Positive | 7 | 11/16/2017 | 30 ^*^ | NEG | ND | ND | ND | ND | N/A |
| 2017 | Positive | 8 | 11/17/2017 | 240 ^*^ | NEG | ND | ND | ND | ND | N/A |
| 2017 | Positive | 9 | 11/23/2017 | 54 ^*^ | Positif | ND | ND | ND | ND | N/A |
| 2017 | Positive | 10 | 11/23/2017 | 120 ^*^ | NEG | ND | ND | ND | ND | N/A |
| 2017 | Positive | 11 | 11/28/2017 | 142 ^*^ | NEG | ND | ND | ND | ND | N/A |
| 2017 | Positive | 12 | 12/4/2017 | 100 ^*^ | NEG | ND | ND | ND | ND | N/A |
| 2017 | Positive | 13 | 12/4/2017 | 80 ^*^ | NEG | ND | ND | ND | ND | N/A |
| 2017 | Positive | 14 | 12/14/2017 | 100 ^*^ | NEG | ND | ND | ND | ND | N/A |
| 2018 | Positive | 15 | 2/5/2018 | 240 ^*^ | Positif | ND | ND | ND | ND | N/A |
| 2018 | Positive | 16 | 2/27/2018 | 60 ^*^ | NG | ND | ND | ND | ND | N/A |
| 2018 | Positive | 17 | 2/27/2018 | 8 ^*^ | Positif | ND | ND | ND | ND | N/A |
| 2018 | Positive | 18 | 2/27/2018 | 44 ^*^ | NEG | ND | ND | ND | ND | N/A |
| 2018 | Positive | 19 | 2/27/2018 | 22 ^*^ | NG | ND | ND | ND | ND | N/A |
| 2018 | Positive | 20 | 2/27/2018 | 54 ^*^ | NEG | ND | ND | ND | ND | N/A |
| 2018 | Positive | 21 | 2/27/2018 | 48 ^*^ | NEG | ND | ND | ND | ND | N/A |
| 2018 | Positive | 22 | 2/27/2018 | 28 ^*^ | NEG | ND | ND | ND | ND | N/A |
| 2018 | Positive | 23 | 2/27/2018 | 50 ^*^ | NEG | ND | ND | ND | ND | N/A |
| 2018 | Positive | 24 | 2/27/2018 | 108 ^*^ | NEG | ND | ND | ND | ND | N/A |
| 2018 | Positive | 25 | 2/27/2018 | 220 ^*^ | NEG | ND | ND | ND | ND | N/A |
| 2018 | Positive | 26 | 2/27/2018 | 32 ^*^ | NEG | ND | ND | ND | ND | N/A |
| 2018 | Positive | 27 | 2/27/2018 | 88 ^*^ | NEG | ND | ND | ND | ND | N/A |
| 2018 | Positive | 28 | 2/28/2018 | 60 ^*^ | NEG | ND | ND | ND | ND | N/A |
| 2018 | Positive | 29 | 2/28/2018 | 10 ^*^ | NEG | ND | ND | ND | ND | N/A |
| 2018 | Positive | 30 | 2/28/2018 | 36 ^*^ | NEG | ND | ND | ND | ND | N/A |
| 2018 | Positive | 31 | 2/28/2018 | 66 ^*^ | NEG | ND | ND | ND | ND | N/A |
| 2018 | Positive | 32 | 2/28/2018 | 210 ^*^ | NEG | ND | ND | ND | ND | N/A |
| 2018 | Positive | 33 | 3/2/2018 | 50 ^*^ | NEG | ND | ND | ND | ND | N/A |
| 2017-2018 | Negative | 34-39 | 10/26/2017-2/27/2018 | < 0.6 IU/mL | < 0.80 IU/mL | ND | ND | ND | ND | N/A |

**^a^** This new variant has a black indicator, enhancing contrast when tested using whole blood, a previous issue with the pink indicator of the serum-variant test. **^b^** “Year of conventional test” refers to year conventional testing was performed in a reference laboratory in the U.S., France, or Morocco. When an individual has *T. gondii* infection, antibody to the parasite persists. Thus, if a person had anti-*Toxoplasma* antibodies in the past, they retain antibody across time. Therefore, previous testing from the NCCCTS cohort (almost always performed by the Palo Alto reference laboratory) that is positive indicates that the individual Is infected/seropositive. Concurrent, standard laboratory testing was with ARCHITECT system in Lyon, France for U.S. samples and Platelia^TM^ system in Rabat, Morocco. **^c^** An IgG dye test is considered negative for values <1:16 and positive for values ≥1:16 [1]. **^d^** An IgM ELISA performed with serum is considered negative for values 0.0-1.6, equivocal for values 1.7-1.9, and positive for values ≥2.0 in serum [2]. An IgM ISAGA is positive for values ≥3 [3]. **^e^** An IgA ELISA for patients >6 months of age is considered negative for values 0.0-1.4, equivocal for values 1.5-2.0, and positive for values ≥2.1, and an IgA ELISA for patients <6 months of age is considered negative for values 0.0-0.9 and positive for values ≥1.0 [4]. **^f^** High avidity signifies that infection occurred more than 4 months ago [5].**^*^** Serology not performed at Palo Alto Medical Foundation *Toxoplasma* Serology Laboratory. These were performed at the reference laboratory in Lyon, France using the ARCHITECT system (for United States samples) or in the Moroccan reference laboratory in Rabat using the Platelia^TM^ system. References for test performance are available in the literature [6,7].

**Literature Cited:**

1. Sabin AB, Feldman HA. Dyes as Microchemical Indicators of a New Immunity Phenomenon Affecting a Protozoon Parasite (Toxoplasma). Science **1948**; 108:660–663.

2. Naot Y, Remington JS. An Enzyme-Linked Immunosorbent Assay for Detection of IgM Antibodies to Toxoplasma gondii: Use for Diagnosis of Acute Acquired Toxoplasmosis. J Infect Dis **1980**; 142:757–766.

3. Desmonts G, Naot Y, Remington JS. Immunoglobulin M-immunosorbent agglutination assay for diagnosis of infectious diseases: diagnosis of acute congenital and acquired Toxoplasma infections. J Clin Microbiol **1981**; 14:486–491.

4. Stepick-Biek P, Thulliez P, Araujo FG, Remington JS. 19A Antibodies for Diagnosis of Acute Congenital and Acquired Toxoplasmosis. J Infect Dis **1990**; 162:270–273.

5. Pelloux H, Brun E, Vernet G, et al. Determination of anti–Toxoplasma gondii immunoglobulin G avidity: adaptation to the vidas system (bioMérieux). Diagn Microbiol Infect Dis **1998**; 32:69–73.

6. Gay-Andrieu F, Fricker-Hidalgo H, Sickinger E, et al. Comparative evaluation of the ARCHITECT Toxo IgG, IgM, and IgG Avidity assays for anti-Toxoplasma antibodies detection in pregnant women sera. Diagn Microbiol Infect Dis **2009**; 65:279–287.

7. Hofgärtner WT, Swanzy SR, Bacina RM, et al. Detection of immunoglobulin G (IgG) and IgM antibodies to Toxoplasma gondii: evaluation of four commercial immunoassay systems. J Clin Microbiol **1997**; 35:3313–3315.
